# Interference of the positioning of nickel, chromium and cobalt in the results of patch tests^☆^

**DOI:** 10.1016/j.abd.2024.03.016

**Published:** 2024-12-10

**Authors:** Ida Duarte, Rosana Lazzarini, Mariana Haffner, Julia P. Silva

**Affiliations:** a Department of Medicine, Faculty of Medical Sciences, Santa Casa de São Paulo, São Paulo, SP, Brazil; b Department of Medicine, Dermatology Clinic, Santa Casa de São Paulo, São Paulo, SP, Brazil

**Keywords:** Allergic contact dermatitis, Dermatitis, contact, Metals, patch tests

## Abstract

**Background:**

The positioning of substances, which are co-sensitizers and/or with a tendency to cross-react, is not considered in the technique when applying patch tests (PT).

**Objective:**

To investigate the interference of the positioning of nickel (Ni), chromium (Cr) and cobalt (Co) in patch tests (PT) results, when applied close or distant from each other.

**Methods:**

PT were performed in patients suspected of allergic contact dermatitis (ACD), using the standard battery (SB), with substances showing a tendency towards cross-react and co-sensitizers applied far apart, and an additional battery (AB) with the metals placed close to each other. For tabulation purposes, only the 96-h reading was considered.

**Results:**

Of the 86 tested patients, 33 (38%) had negative testing for metals and 53 (62%) had one or more positive (+) tests for Ni, Cr and/or Co. Concordant results in both tests (SB/ AB) occurred in 18/53 (34%) and 35/53 (66%) had discordant results. Regarding the SB, of the 159 tests with metals (53 patients, three metals), 57 tests were (+) and 102 (-). In the AB, 87 tests were (+) and 72 (-), a statistically significant difference (p < 0.05). Of the 57 (+) tests in the SB, 35 were for Ni, 18 for Co and four for Cr. In AB the number of (+) tests was 87, with 45 (+) tests for Ni, 35 for Co and seven for Cr. The difference in the number of positive tests between the two batteries was statistically significant for Co and Ni.

**Study limitations:**

The number of cases.

**Conclusion:**

The results showed that the positioning of the metals interferes with the PT results and should be considered as part of the PT application technique.

## Introduction

Allergic contact dermatitis (ACD) caused by metals has as its main agents nickel (Ni), chromium (Cr) and/or cobalt (Co).^1^, ^2^, ^3^, ^4^

The distribution of ACD to metals in each country is heterogeneous since the manifestation on this dermatosis is influenced by atopy, gender, age, ethnicity, environmental exposure, genetic, geographic, socio-environmental and occupational factors.^1^, ^2^, ^5^

In the USA, approximately 18% of the population is sensitized to Ni, which is the main sensitizer; the frequency of sensitization to Co is 7.3% and 1.5% to Cr.^2^ In Europe, the prevalence of allergy to Ni occurs in approximately 19% of the population, to Co 3.6% and 9.6% to Cr.^1^ In Brazil, population studies have shown that approximately 28% of the population is sensitized to Ni, 10.5% to Co and 11% to Cr.^3^, ^4^ Thus, ACD to metals is common in all populations, whether or not related to occupational activities.^5^, ^6^, ^7^

ACD is diagnosed through clinical history, presentation and patch tests (PT), the latter being the main laboratory method for its identification. Obtaining accurate test results depends on their indication, application technique and adequate reading.

There are few studies on the interference of the positioning of the substances in relation to their co-sensitizers or others substances with a tendency towards cross-reaction with each other.^8^, ^9^, ^10^ Ni, Cr and Co do not belong to the same chemical group; therefore, there is no induction of cross-reaction between these substances. However, because they are present together in several materials, they can act as co-sensitizers. The concept of co-sensitizer refers to chemicals that act together to increase sensitivity to a specific phenomenon.^11^ In the case of ACD, Ni, Co and Cr, when present in the same material, can induce sensitization or enhance each others immune reaction.

As previously mentioned, metals are the main sensitizers in the general population. Therefore, accurate PT results are essential for the etiological diagnosis of ACD, as well as the recommendations given to the patient.

## Objective

To investigate the interference of the positioning of Ni, Co and Cr in the results of patch tests, when applied close or far from each other.

## Method

The research project was approved by the Research Ethics Committee (REC) of Santa Casa de São Paulo, as a prospective study, under Counsel number 4,713,172 on 05/14/2021.

During the 12-month period (November 2021 to October 2022), patients treated in the allergy sector of the Dermatology Clinic of a healthcare service, with a diagnostic hypothesis of ACD and indication for PT, were selected.

The standard battery (SB) of PT, consisting of 30 substances, was applied to the left side of the back region of these patients. This PT battery is standardized with co-sensitizing and cross-reactive substances applied far apart from each other. The SB was applied using FINN Chambers (FDA Allergenic), with 10 substances on each plate, placed one below the other. Potassium bichromate (Cr) corresponded to number 5, Co to number 11 and Ni to number 27. Thus, in the SB used, the metals were applied far apart from each other, in different chambers.

The patients were also concomitantly tested with Ni, Co and Cr close to each other, constituting an additional battery (AB) with three components, applied to the right side of the back region. The metals were applied using FINN Chambers, cut so that they contained only three aluminum plates, where the metals were placed.

Two readings of the tests were performed, the first 48 hours after application and the second after 96 hours. For tabulation purposes, only the 96-h reading was considered and all tests with intensity 1+, 2+ or 3+ were tabulated.

Since in the SB, substances with a tendency towards cross-reaction and/or co-sensitizers are applied far from each other, tests that were positive to the other battery components did not interfere with the metal tests.

The results of the metal tests obtained in the SB and AB were tabulated and compared with each other. Regarding the statistical analysis, the Chi-Square test was used, aiming to compare the proportions of the results.

## Results

During the 12-month period (October 2021 to November 2022), 86 patients underwent patch testing, 23 men and 63 women, 58 white and 28 non-white individuals (10 black; 17 brown and 1 Asian). Patients ages ranged from 7 to 85 years, with a mean age of approximately 45.5 years. Patients professions are listed in Table 1 and the locations of the dermatosis are listed in Table 2.

**Table 1:** Distribution of patients submitted to patch testing according to their profession ‒ 86 patients.

Profession	n	%
Office worker/student	23	27
Health workers	18	21
Homemaker	19	22
Retired worker	14	16,5
Mason/painter/maintenance worker	07	08
Seamstress	02	2,5
Butcher	01	01
Hairdresser	01	01
Cook	01	01
Total	86	100

**Table 2:** Main ACD locations – 86 patients.

Location	n	%
Hands	28	33
Lower limbs	28	33
Upper limbs	26	30
Cephalic segment	21	25
Trunk	20	23
Feet	15	17
Abdomen	7	8
Total^a^	145	

a Some patients had lesion in more than one location.

Of the 86 tested patients, 33 (38%) had negative tests for Ni, Co and Cr. The remaining 53 patients (62%) had one or more positive tests for the metals. Of these, 18/53 (34%) showed concordant results in both tests (SB and AB). The remaining 35/53 (66%) showed discordant results in the two tests (Table 3).

**Table 3:** Distribution of the 86 patients according to the results of the metal patch testing in the standard battery and the additional battery.

Results of the metal patch testing	n	%		n	%
Patients with negative tests	33	38			
Patients with positive tests	53	62	Concordant results (SB and AB)	18/53	34
			Discordant results (SB and AB)	35/53	66
Total	86	100			

SB, Standard Battery; AB, Additional Battery.

Table 4 depicts the number of positive tests for Ni, Co and Cr in the two tests.

**Table 4:** Number of positive metal tests in 53 patients.

	Nickel		Cobalt		Chromium	
	SB	AB	SB	AB	SB	AB
Concordant results SB and AB (18)	9	9	6	6	3	3
Discordant results SB and AB (35)	26	36	12	29	1	4
Total	35^a^	45^b^	18^a^	35^b^	4^a^	7^b^

a SB, Standard Battery = 57 positive tests.

b AB, Additional Battery = 87 positive tests.

Regarding the patients who tested positive for metals (53/86), each one underwent three metal contact tests in the SB and three other tests that comprised the AB of tests. Thus, there were 159 tests related to Ni, Co and Cr (53 × 3) in each battery.

In the SB, of the 159 tests related to metals, 57 were positive and 102 were negative. In the AB, there were 87 positive and 72 negative tests. The differences found in the results between the two batteries were statistically significant (p < 0.05; Table 5).

**Table 5:** Results of epicutaneous metal patch testing in test batteries in 53 patients.

Metals	Positive tests		Negative tests		Total
Standard battery	57	36%	102	64%	159
Additional battery	87	55%	72	45%	159
Total	144		174		318

p = 0.00007.

Regarding the 57 positive results of the standard battery, 35 were positive for Ni, 18 for Co and 4 for Cr. In the additional battery, the number of positive tests was 87, of which 45 were positive for Ni, 35 for Co and seven for Cr (Table 6, Table 7, Table 8).

**Table 6:** Test results for Nickel in 53 patients.

Nickel	Positive tests		Negative tests		Total
Standard battery	35	66%	18	34%	53
Additional battery	45	85%	8	15%	53
Total	80		26		106

p = 0.02.

**Table 7:** Test results for Cobalt in 53 patients.

Cobalt	Positive tests		Negative tests		Total
Standard battery	18	34%	35	66%	53
Additional battery	35	66%	18	34%	53
Total	53		53		106

p = 0,0009.

**Table 8:** Test results for Chromium in 53 patients.

Chromium	Positive tests		Negative tests		Total
Standard battery	4	5%	49	95%	53
Additional battery	7	13%	46	87%	53
Total	11		95		106

p = 0.33.

After applying the Chi-square test to the test results for each metal and comparing the results in the two batteries, a statistically significant difference was observed related to the number of positive tests. In other words, applying the tests to metals close to each other significantly interfered with the results for Ni and Co.

## Discussion

The epidemiological data in this study demonstrated that the group was similar to those in other studies: a predominance of white female individuals, with a mean age of 45 years.^1^, ^2^, ^3^, ^4^

The majority of the patients (62%) tested positive for one or more metals, with Ni being the main sensitizer in both batteries, followed by Co and Cr. These results demonstrate the relevance of metal sensitization, especially Ni and Co, components of several products present in people daily lives.^12^, ^13^, ^14^, ^15^, ^16^, ^17^ Ni, Co and Cr are responsible for occupational and non-occupational ACD.

The results obtained in the two batteries showed interference between them, due to the positioning of the Ni, Co and Cr applied in the patch tests. The element Ni showed an additional ten positive tests, in the results of the SB, when it was applied close to Co and Cr (66% of positive tests in the SB and 85% in the AB), with a statistically significant difference (p < 0.05). The same occurred with Co, with a difference of 17 tests between the SB (34% positive tests) and the AB (66% positive tests), also with statistical significance (p < 0.05). Cr showed four positive tests (5%) in the SB and seven in the AB (13%). The small number of positive tests did not allow a significant analysis.

The differences in positive tests for metals that occurred between the two batteries (SB and AB) probably occurred due to the excited skin syndrome, where co-sensitizing substances or those associated with cross-reaction with each other, induce the excited skin syndrome when placed close to each other.^8^, ^9^

Ni, Co and Cr are metals known as common allergens, found in several materials present in people daily lives and can act as co-sensitizers, enhancing the immune response to these components. Riedel et al.^11^ demonstrated that in the immune reaction caused by exposure to Ni, T-cell receptors can be stimulated by Co, justifying co-sensitization. Pistoor et al.^18^ showed interference in the reaction between Ni, copper and palladium.

The application of patch tests to Ni, Co and Cr, when placed close together, can induce co-sensitization, leading to an increase in positive tests for these metals. In practice, it is common for patients to show positive tests for two or all three metals.^19^, ^20^

Ni, Co and Cr are common sensitizers in several population groups, as they are common components of products and materials, present in daily routine and in several professional activities. The results of patch tests to these substances has a relevant impact in the routine of patients as well as in their professional activity.

In conclusion, the positioning of the metals (Ni, Co and Cr) during the application of patch tests is an important aspect for their interpretation, aiming to obtain accurate results and provide correct recommendations for the patients.

## Financial support

FAP2021/2023 FCMSCSP.

## Authors' contributions

Ida Duarte: Study design; Collection of data; drafting and editing of the manuscript.

Rosana Lazzarini: Patch testing; critical review of the manuscript.

Mariana Haffner: Patch testing; critical review of the manuscript.

Julia P Silva: Data tabulation; Literature survey.

## Conflicts of interest

None declared.
